# Data-driven approach for tailoring facilitation strategies to overcome implementation barriers in community pharmacy

**DOI:** 10.1186/s13012-021-01138-8

**Published:** 2021-07-19

**Authors:** Lydia Moussa, Shalom Benrimoj, Katarzyna Musial, Simon Kocbek, Victoria Garcia-Cardenas

**Affiliations:** 1 Graduate School of Health, Faculty of Health, University of Technology Sydney, PO Box 123, Broadway, Sydney, New South Wales 2007 Australia; 2 Pharmaceutical Care Research Group, Faculty of Pharmacy, University of Granada, Campus Universitario de Cartuja s/n, 18071 Granada, Spain; 3 Advanced Analytics Institute, School of Computer Science, Faculty of Engineering and IT, University of Technology Sydney, PO Box 123, Broadway, Sydney, New South Wales 2007 Australia; 4 Advanced Analytics Institute, School of Software, Faculty of Engineering and IT, University of Technology Sydney, PO Box 123, Broadway, Sydney, New South Wales 2007 Australia

**Keywords:** Change facilitation, Implementation factors, Determinants, Tailored interventions, Facilitation strategies, Pharmacy practice, Change management, Organisational change, Machine learning, Random forest

## Abstract

**Background:**

Implementation research has delved into barriers to implementing change and interventions for the implementation of innovation in practice. There remains a gap, however, that fails to connect implementation barriers to the most effective implementation strategies and provide a more tailored approach during implementation. This study aimed to explore barriers for the implementation of professional services in community pharmacies and to predict the effectiveness of facilitation strategies to overcome implementation barriers using machine learning techniques.

**Methods:**

Six change facilitators facilitated a 2-year change programme aimed at implementing professional services across community pharmacies in Australia. A mixed methods approach was used where barriers were identified by change facilitators during the implementation study. Change facilitators trialled and recorded tailored facilitation strategies delivered to overcome identified barriers. Barriers were coded according to implementation factors derived from the Consolidated Framework for Implementation Research and the Theoretical Domains Framework. Tailored facilitation strategies were coded into 16 facilitation categories. To predict the effectiveness of these strategies, data mining with random forest was used to provide the highest level of accuracy. A predictive resolution percentage was established for each implementation strategy in relation to the barriers that were resolved by that particular strategy.

**Results:**

During the 2-year programme, 1131 barriers and facilitation strategies were recorded by change facilitators. The most frequently identified barriers were a ‘lack of ability to plan for change’, ‘lack of internal supporters for the change’, ‘lack of knowledge and experience’, ‘lack of monitoring and feedback’, ‘lack of individual alignment with the change’, ‘undefined change objectives’, ‘lack of objective feedback’ and ‘lack of time’. The random forest algorithm used was able to provide 96.9% prediction accuracy. The strategy category with the highest predicted resolution rate across the most number of implementation barriers was ‘to empower stakeholders to develop objectives and solve problems’.

**Conclusions:**

Results from this study have provided a better understanding of implementation barriers in community pharmacy and how data-driven approaches can be used to predict the effectiveness of facilitation strategies to overcome implementation barriers. Tailored facilitation strategies such as these can increase the rate of real-time implementation of innovations in healthcare, leading to an industry that can confidently and efficiently adapt to continuous change.

**Supplementary Information:**

The online version contains supplementary material available at 10.1186/s13012-021-01138-8.

Contribution to the literature This paper contributes to implementation literature through:

- The use of innovative data-driven approaches to provide predictions of effective tailored facilitation strategies to be used during implementation of innovations.
- The need to tailor change facilitation strategies according to contextual implementation barriers, rather than a one size fits all approach to implementation.
- The identification of ‘real-world’ barriers experienced in community pharmacy during implementation by an external, objective third party such as a change facilitator.
- Increased efficiency and effectiveness of tailored facilitation interventions, as change facilitators can start choosing strategies with the highest predictive resolution rate in relation to the implementation barrier they uncover in practice.
- Raising awareness of, and providing, an approach to understand and overcome implementation barriers for future implementation projects throughout healthcare.

## Background

Governments and health care practitioners share common goals to improve patients’ clinical outcomes, quality of life and the rationale use of medicines [1]. To achieve such goals, there has been an increasing international trend towards the implementation of professional pharmacy services in community pharmacy [2, 3]. Professional pharmacy services vary significantly in their objectives and complexity. These evidence-based services can include the provision of drug information, clinical interventions, screening services, medication management services or preventive care services for patients with chronic conditions, amongst others [4]. In the case of community pharmacy, similarly to other health care settings, the implementation of new services is often challenging, with common gaps between the development and evaluation of services and their implementation in routine practice. To assist in bridging this implementation gap, previous research in pharmacy has identified ‘external support/ assistance’ as a critical factor in the implementation process [5, 6]. Similarly, other studies have demonstrated that with proper external support, pharmacists can make important progress towards achieving their implementation goals [7].

The concept of external support provision to aid implementation has been explored in research and practice environments through change facilitation. For example, the Promoting Action on Research Implementation in Health Services (PARiHS) framework presents successful implementation research as a function of the relationship between evidence, context, and facilitation [8]. Change facilitation has become a key component in supporting teams during the implementation of change in practice [9] and has proven effective across a variety of healthcare settings [10]. A change facilitator can provide support to stakeholders to ‘realise what they need to change and how to make changes to incorporate evidence into practice’ [11]. In addition to the need for external support, implementation research recognises that the process of implementation goes beyond simple dissemination of information, requiring the use of strategies that are more specific to the practice’s settings [12]. Previous research in pharmacy has used change facilitators to deliver such strategies, with the ultimate objective of addressing implementation barriers and increase the number of professional services provided [13, 14]. In this sense, the identification of relevant implementation factors and understanding how they prevent or enable implementation have been highlighted as key in aiding the development and assessment of tailored interventions to assist in the implementation of innovations such as professional services [15].

The concept of tailored interventions has been highlighted in the literature with the realisation that ‘no single strategy appears to be sufficient to drive successful implementation’ [16–18]. There is, however, a need for more innovative methods for assessing and prioritising implementation barriers and tailoring implementation strategies to such barriers [19]. This approach is likely to increase the effectiveness of implementation strategies [19], such as change facilitation. The challenge is that the majority of randomised controlled trials involving facilitation rely on patient or implementation outcomes to evaluate the facilitation’s success [20], without further evaluation of the facilitation process or the impact of specific facilitation strategies. Moreover, the core components of effective change facilitation remain unknown. Determining the potential effectiveness of facilitation strategies in specific contexts and settings such as community pharmacy could add evidence on essential activities required for implementation during the facilitation process and ensure the delivery of tailored, evidence-based strategies in research and practice.

As a way of promoting the implementation of professional services in community pharmacies, the Pharmaceutical Society of Australia (PSA) launched a pharmacy change commercial programme named ‘Health Destination Pharmacy’ (HDP) which was delivered from 2016 to 2018. The primary objective of the programme was to reposition the pharmacist as a healthcare provider and the pharmacy as a healthcare destination [15]. To achieve this objective, change facilitation was used as a key implementation strategy in which participating pharmacies received tailored interventions to facilitate the implementation of professional services. This study aimed to explore implementation barriers identified by change facilitators during this 2-year implementation programme and to predict the effectiveness of facilitation strategies to overcome implementation barriers using machine learning techniques.

## Methods

### Study design and context

This study used a mixed-method approach, which included a qualitative analysis of the barriers and facilitation strategies used by change facilitators during HDP. The study also included a quantitative analysis of the effectiveness (based on predictive resolution percentage) of the facilitation strategies used.

The study was undertaken in community pharmacies across Australia. During the 2-year change programme, change facilitators supported pharmacy teams who signed up for the programme, with the goal of increasing their provision of professional services. The support of change facilitators included (1) individual on-site facilitation visits to the pharmacy every 3 months, (2) identification of implementation barriers preventing the pharmacist and the pharmacy team from successfully increasing their provision of professional services, (3) the provision of tailored change facilitation strategies to overcome the identified implementation barriers, and (4) continuous telephone follow-up and monitoring.

### Change facilitator experience and training

All change facilitators were registered pharmacists with experience in community pharmacy, to ensure their relatability to the pharmacists and teams whom they were supporting during implementation. Change facilitators had varying levels of facilitation and/or coaching expertise, but all attended a 2-day mandatory training prior to their allocation into the pharmacies. The 2-day training included the following:

- Previous pharmacy implementation research [21].
- The use of the Generic Implementation Framework (GIF) [1] to underpin the implementation process.
- Implementation barriers previously identified in the literature and existing frameworks such as the Consolidated Framework for Implementation Research (CFIR) [22], the Theoretical Domains Framework (TDF), [19] and the Integrated Checklist of Determinants of practice (TICD) [23].
- One-on-one coaching strategies using the GROW model [24] and role-playing of the use of such models in practice.
- Group facilitation skills training including strategies to engage teams, brainstorming strategies, conflict management strategies, and communication strategies.
- The use of a participant observation guide and data collection form.

### Data collection

Data collection was undertaken on-site in each participant pharmacy. A participant observation guide was designed based on previous research [1, 25] and was used by all facilitators. This guide was developed to allow facilitators to systematically identify and individually evaluate each pharmacy, identifying the implementation factors operating as barriers. During their facilitation visits, change facilitators used the participant observation guide and interviewed each participant pharmacist in order to gain a deep understanding of relevant implementation factors. Post-visit, change facilitators were required to transfer the following information to the data collection form:

- The identified implementation factors that acted as barriers. The data collection form included a pre-defined list of implementation barriers. Change facilitators could choose the identified barrier from a drop-down list, or they could add a barrier if they could not find an appropriate one from the list. The data collection form also included a section to provide additional qualitative data regarding the identified barrier.
- The facilitation strategies they used to overcome the identified barriers. Change facilitators documented the facilitation strategies provided using qualitative data.
- At which visit they identified each barrier and conducted the facilitation strategy.
- Whether the barrier was resolved or unresolved. Change facilitators indicated whether the barrier was resolved based on the following criteria: (1) if an agreed upon action by the facilitator and the pharmacy team member was completed by the next pharmacy visit or follow-up phone call or (2) if an increase in the provision of professional pharmacy services was a direct result from this strategy.

### Data coding

To ensure data validity, the research project manager reviewed the database following each facilitation visit throughout the 2-year duration of the programme. On completion of the project, the same researcher coded the implementation barriers and the facilitation strategies. Implementation barriers were coded using a pre-defined list based on the CFIR [22], TICD [23] and TDF [22] (Additional file 1). Facilitation strategies were mapped according to those identified from two systematic reviews [9, 26] (Additional file 2). One systematic review looked at facilitation strategies conducted in nursing, from which the Taxonomy of Facilitation Strategies was developed [9], and the second systematic review identified facilitation strategies recorded in randomized controlled trials focusing on the implementation of innovation in healthcare [26].

### Data analysis using the data mining approach random forest

In pursuit of accurate representation of the data collected, a descriptive analysis was initially undertaken to provide a resolution percentage for each of the facilitation strategies recorded. However, if one strategy was only used once and was recorded as having resolved the barrier, this would give it a 100% resolution rate. If another strategy was used in 10 barriers and was recorded as having resolved 9 of the 10 barriers, this would have a resolution rate of 90%. Traditional statistical approaches were therefore deemed inaccurate in adequate representation of the effectiveness of the strategies as it is based purely on the number of times that the strategy was used. For this reason, machine learning with random forest (RF) was used as an alternative predictive approach (Additional file 3). As opposed to the resolution rate from the statistical analysis approach, when using RF, a predictive resolution percentage (PRP) is given to each of the facilitation strategies in relation to the barriers that were resolved by that particular strategy. An algorithm is used to rank the facilitation of strategies in order of PRP’s. The higher the PRP, the more likely that the strategy is predicted to overcome the related barrier. It should be noted that prediction methods are not assumptions, but are calculated based on the extrapolation of actual data collected by the change facilitators. Random forest was used as a supervised classification method for predicting effective strategies for all barriers. Supervised classification uses data recorded by the change facilitators, to train a machine learning model to predict future outcomes. All strategies in the dataset were labelled with an outcome: ‘strategy works’ (i.e. the barrier was resolved) or ‘strategy does not work’ (i.e. the barrier was unresolved). RF classification algorithm was chosen, due to its popularity in the industry, explainability and accuracy, and its enhanced resistance to overfitting than the standard decision tree models (i.e. not generalising well to new instances). For example, Khalilia et al. [27] used RF to predict disease risk of individuals by analysing their medical diagnosis, RF has also been used to predict novel risk genes such as lupus nephritis [28] and has been proposed to predict risk of type II diabetes [29]. RF combines great numbers of decision trees trained randomly and equally from a given dataset. To ensure the machine learning algorithm used increased the PRP accuracy, 10-fold cross-validation [26] technique was adopted, where data was randomly split into ten groups (folds). For each group, this given group was taken as a test dataset and the remaining nine groups as a training set. Then, a model was fitted on a training dataset and was evaluated on the test set. The evaluation score was kept and the model was discarded. This procedure was then repeated ten times. To get a performance of a model, the average of all ten evaluation scores was taken.

### Reporting of the most common implementation barriers and strategies

In order to help prioritise the focus on the most common barriers and the facilitation strategies used to overcome them, Pareto’s principle was used. Pareto’s principle states that, for many events, roughly 80% of the effects come from 20% of the causes. This principle has been proven effective in organisational decision making [30]. For this reason, the results reported in this paper focus on the top 20% of barriers, according to the frequency in which they appeared in the database.

## Results

Nineteen pharmacies participated in the change programme. They were located across Australia and ranged in the number of prescriptions dispensed per year from a minimum of 23,954 to a maximum of 223,269 with an average of 93,239 prescriptions dispensed per year. The number of employees in the participant pharmacies ranged from a minimum of two to a maximum of 46 staff members. Six change facilitators were allocated to the 19 pharmacies based on the geographical location of the change facilitator in accordance to the pharmacy.

One thousand one hundred thirty-one data points (i.e. total number of barriers identified and associated facilitation strategies provided in the participant pharmacies during the 2-year programme) were recorded. The random forest algorithm used was able to provide 96.9% accuracy in predicting the most effective strategies to overcome specific barriers to implementation. Table 1 showcases the strategies used to address the top 20% (n=7) most common implementation barriers identified by change facilitators across the 2-year programme.

**Table 1:** Facilitation categories used to overcome common implementation barriers in community pharmacy

Most common barriers to implementing professional services in community pharmacy^	Strategy categories* used by change facilitators to overcome implementation barriers	The predictive resolution percentage of the strategy category resolving the barrier (PRP)^a^
An inability to plan for change (*n*=184)	Engage stakeholders by creating ownership of the change	84%
	Equip stakeholders with training	83%
	Adapt area of focus to meet change needs	81%
A lack of internal supporters of the change (*n*=128)	Engage stakeholders by creating ownership of the change	78%
	Empower stakeholders to develop objectives and solve problems	73%
	Create buy-in of the change amongst stakeholders	58%
A lack of knowledge and experience related to the change (*n*=84)	Create a collaborative environment conducive of change	99%
	Equip stakeholders with training	93%
A lack of monitoring and feedback of the change (*n*=61)	Feedback implementation progress	99%
	Ensure continuous monitoring of implementation measures	68%
A lack of individual alignment with the change (*n*=49)	Encourage participation and facilitate discussions amongst stakeholders	99%
	Empower stakeholders to develop objectives and solve problems	83%
	Create buy-in of the change amongst stakeholders	83%
Undefined change objectives and lack of objective feedback (*n*=46)	Engage stakeholders by creating ownership of the change	82%
	Empower stakeholders to develop objectives and solve problems	81%
	Communicate the change to stakeholders	63%
A lack of time (*n*=43)	Adapt area of focus to meet change needs	79%
	Empower stakeholders to develop objectives and solve problems	62%

^A total of 1131 barriers were identified across the 19 pharmacies throughout the 2-year period

*The strategy categories were adapted from the taxonomy of facilitation strategies by Dogherty et al.

111 facilitation strategies were coded into 16 facilitation categories; the strategies within each of the above-mentioned categories can be found in Table 2

^a^Predictive resolution percentage is based on a data-driven approach named decision forest which used data collected by change facilitators indicating whether each strategy resolved the barrier or not

### The top 20% most common implementation barriers

‘*An inability to plan for change*’ was the most commonly identified barrier. It was identified 184 times across 16 of the 19 pharmacies. This implementation factor is described by the TICD checklist as ‘the extent to which the targeted healthcare professionals are able to plan necessary changes in order to adhere’. To overcome this barrier, the change facilitators used strategies to (1) engage stakeholders by creating ownership of the change, which had a predictive resolution percentage (PRP) of 84.23%; (2) equip stakeholders with training (PRP=83.30%); (3) adapt area of focus to meet change needs (PRP=81.17%); and (4) empower stakeholders to develop objectives and solve problems (PRP=80.64%).

‘*A lack of internal supporters to change*’ also known as internal change resistance was identified as a barrier 128 times across 18 of the 19 pharmacies. The TICD checklist describes this barrier as a lack of ‘support provided by the staff members for the implementation of the change’. To overcome this barrier, the change facilitators used strategies to (1) engage stakeholders by creating ownership of the change (PRP= 78.29%), (2) empower stakeholders to develop objectives and solve problems (PRP=73.44%) and (3) create buy-in of the change amongst stakeholders (PRP=57.90%).

‘*A lack of knowledge and experience*’ was identified as a barrier 84 times across 18 of the 19 pharmacies. The TDF describes this implementation factor as ‘the extent to which the targeted individuals have skills, knowledge and experience that they need to adhere’. When this implementation factor became a barrier, i.e. a lack of knowledge and experience, the change facilitators used strategies to (1) create a collaborative environment conducive to change (PRP= 99.80%) and (2) equip stakeholders with training (PRP=93.44%).

‘*A lack of monitoring and feedback*’ was identified as a barrier 61 times across 14 of the 19 pharmacies. The TICD checklist explains this as ‘the extent to which monitoring and feedback are needed at an organisational level and available to sustain necessary changes’. When a lack of monitoring and feedback was identified by the change facilitators as a barrier, they used strategies to (1) feedback progress of implementation measures (PRP= 99.12%) and (2) ensure continuous monitoring of implementation measures (PRP= 68.09%).

‘*A lack of individual alignment with the change*’ was identified as a barrier 49 times across 14 out of the 19 pharmacies. The CFIR defines this as ‘the degree of tangible fit between meaning and values attached to the change by involved individuals’ own norms, values, perceived risks and needs.’ When there was a lack of individual alignment with the change, the change facilitators used strategies to (1) ensure stakeholders contribute to the change (PRP=98.79%), (2) empower stakeholders to develop objectives and solve problems (PRP= 83.13%), (3) create a case for change (PRP=82.86%) and (4) engage stakeholders by creating ownership of the change (PRP= 49.38%)

‘*Undefined change objectives and lack of objective feedback*’ was identified as a barrier 46 times across 16 of the 19 pharmacies. The TICD checklist explains this as ‘the degree to which implementation objectives have been defined, communicated and achieved by the members of the team’. To overcome this barrier, change facilitators used strategies to (1) ‘engage stakeholders by creating ownership of the change’ (PRP= 82.33%), (2) ‘empower stakeholders to develop objectives and solve problems’ (PRP= 80.55%) and (3) ‘communicate the change to stakeholders’ (PRP=62.83%)

‘*A lack of time*’ was identified as a barrier 43 times across 15 out of the 19 pharmacies. To overcome this barrier, change facilitators used strategies to ( 1) ‘adapt area of focus to change requirements’ (PRP=79.09%) and (2) ‘empower stakeholders to develop objectives and solve problems’ (PRP=62.25%).

Whilst Table 1 showcases the most common barriers (n=7) identified and the facilitation categories (n=10) used to overcome these barriers, Table 2 breaks down the most effective categories (*n*=10) to showcase the specific strategies within each of the categories and the barriers which these categories overcame. The facilitation category that was used to resolve the most barriers was ‘to empower stakeholders to develop objectives and solve problems’. This category was used to overcome six barriers including ‘an inability to plan for change’, a ‘lack of internal supporters for the change’, a ‘lack of individual alignment to the change’, ‘undefined change objectives’, a ‘lack of objective feedback’, and a ‘lack of time’. The facilitation category with the lowest PRP was ‘communicate the change to stakeholders’. This category was used to overcome the implementation barrier of ‘undefined change objectives’ and ‘lack of objective feedback’ with a PRP of 62.83%.

**Table 2:** Facilitation strategies used by change facilitators to overcome common implementation barriers in community pharmacy

Strategy category to overcome barrier*	Facilitation strategies within category	Most common barriers overcome using this strategy category (PRP)^a^
**Empower stakeholders to develop objectives and solve •lems**	• Stimulate critical inquiry/ critical reflection • Utilise think-aloud process • Utilise brainstorming techniques • Outlining opportunities presented by change • Conduct a needs analysis • Conduct a Strength, Weaknesses, Opportunities and Threats (SWOT) analysis • Use prioritisation techniques • Introduce goal-setting (SMART goals) • Use consensus-building/shared decision-making • Providing solutions/advice • Create/ recommend the creation of a monthly or annual plan • Ensure win/win goals (mutually beneficial solutions) • Use an action planner tool • Use a mind-mapping tool • Discuss/ outline best practices	• An inability to plan for change (80.64%) • A lack of internal supporters of the change (73.44%) • A lack of individual alignment with the change (83.13%) • Undefined change objectives and lack of objective feedback (80.55%) • A lack of time (62.25%)
**Engage stakeholders by creating ownership of the change**	• Establish/ allocate roles • Delegate responsibilities • Allocate primary champion and/or supporting champions • Define key performance indicators • Ask for commitment to the agreed changes • Encourage collaboration and teamwork • Recommend or aid in conducting a performance review • Allocate roles based on skills/ interests • Emphasise the importance of delegating	• An inability to plan for change (84.23%) • A lack of internal supporters of the change (78.29%) • A lack of individual alignment with the change (49.38%)
**Equip stakeholders with training**	• Provide/recommend skills/technical training • Provide knowledge training • Conduct/ recommend role-playing/role modelling • Bringing subject matter expert • Refer to external formal education/training • Using case studies • Use a staff scoping and training tool • Encourage discussion of training topic as a group • Create/adapt training plan • Determine training gaps • Encourage self-learning (e.g reading of journals)	• A lack of knowledge and experience related to the change (93.44%) • An inability to plan for change (83.30%)
**Adapt area of focus to meet change needs**	• Adapt task allocations by creating a roster to align with change • Improve workflow by adapting layout to cater for change • Adapt vision/mission to align for change • Review roles to align with change requirements • Create time-tabling (annual, monthly or weekly time tables) • Adapt business strategy plan to the change • Adapt image of organisation towards new changes • Create/adapt communication plan to new changes • Adapt process/procedures to new changes • Encourage regular communication amongst participants to ensure everyone is aligned to new changes	• An inability to plan for change (81.17%) • A lack of time (79.09%)
**Create buy-in amongst stakeholders**	• Ask about individual concerns regarding the change • Address specific individual concerns related to the change • Motivate group/individuals using stories • Compare audit results to network benchmarking results • Emphasise enhanced customer outcomes as opposed to poor practice • Outline negative impacts to lack of implementation (using evidence/opinion) • Outlining benefits of implementation (using evidence/opinion)	• A lack of individual alignment with the change (82.86%) • A lack of internal supporters of the change (57.90%)
**Create a collaborative environment conducive to change**	• Organise or conduct meetings (face-to-face) • Lead virtual meeting (coach present digitally e.g. webinar or skype)	• A lack of knowledge and experience related to the change (99.80%)
**Feedback progress of implementation measures**	• Provide constructive feedback • Acknowledge success/recognise/celebrate achievements • Provide ongoing encouragement	• A lack of monitoring and feedback regarding the change (99.12%)
**Ensure stakeholders contribute to the change**	• Acknowledge ideas • Encourage knowledge/experience sharing • Involve others in the change process • Acknowledge the importance of individuals’ roles	• A lack of individual alignment with the change (98.79%)
**Ensure continuous monitoring of implementation measures**	• Monitor financial impact • Measure and monitor customer outcomes • Monitor service provision • Monitor Staff measures • Emphasise ongoing monitoring by stakeholders • Monitor agreed upon plan/objectives • Display progress chart	• A lack of monitoring and feedback of the change (68.09%)
**Communicate the change to stakeholders**	• Inform the entire group of the change and objectives verbally • Inform individuals of the change and objectives verbally • Inform using a visual display such as poster • Inform using a written document (email, letter, etc).	• Undefined change objectives and lack of objective feedback (62.83%)

*The strategy categories were adapted from the taxonomy of facilitation strategies (Dogherty et al.)

^a^ PRP is the predictive resolution percentage is based on random forest which uses data collected by change facilitators indicating whether the extent which the strategy is predicted to resolve the barrier

## Discussion

This study has shown change facilitation, not only as an intervention to aid in the implementation of innovation in practice, but also as a way to unearth implementation barriers and provide more tailored facilitation strategies to overcome such barriers within a specific industry such as community pharmacy.

When surveyed or questioned regarding barriers to implementation, healthcare professionals may not provide an accurate representation of the true barriers in practice, but a perception or assumption of the barrier [31]. Implementation research has also stressed the need to focus on what people do rather than what they believe or intend [32]. Having an external, objective third party, such as a change facilitator, provides an alternative view of the barriers with the aim of ‘identifying areas of improvement' [33]. An example of this is that the challenge often posed by pharmacy teams when asked to implement innovations such as professional services is a ‘lack of time’ [31, 34, 35]. Whilst a ‘lack of time’ was raised as a barrier 43 times across the 19 pharmacies over the 2-year programme, however, in this study, this was not the most common barrier as recorded by change facilitators.

As identified in this study, the most frequently occurring barrier was the ‘inability to plan for change’, appearing in 16 out of the 19 pharmacies. The consistency of this barrier in pharmacies across Australia alludes to an overarching inability for pharmacists to adapt to change. Such a challenge has previously been highlighted with an emphasis for pharmacy education to address this barrier to implementation and build pharmacy students’ ability to adapt to change [36]. The ability to plan for change allows pharmacy teams to become more adaptable, which is a major factor in ensuring the sustainability of innovation such as professional services in community pharmacy [37]. For pharmacists in practice, this can be addressed by governing pharmacy bodies and by pharmacy owners equipping their teams with the right capabilities to plan for change and become more adaptable, this is crucial because if ‘pharmacy practice is to survive as an active participant in emerging healthcare systems, pharmacy practice must change along with the rest of health care’ [38].

It is important to note that the change facilitation categories, with the highest PRP, used to overcome the ‘inability to plan for change’, included helping teams ‘engage stakeholders by creating ownership of the change’, ‘equipping stakeholders with training’, helping teams ‘adapt area of focus to meet change needs’, and ‘empowering stakeholders to develop objectives and solve problems’. Strategies in these categories included ‘stimulating critical inquiry’, ‘utilising brainstorming techniques’, ‘utilising goal-setting’, ‘using consensus-building’, ‘shared decision making’ and ‘ensuring mutually beneficial solutions’. In addition, when looking at the facilitation category that resolved the most barriers, this was ‘to empower stakeholders to develop objectives and solve problems’—another category aimed at empowering teams to solve their own challenges and build their own plan for change.

A growing body of evidence highlights that performance can be enhanced when actions are taken that result in empowering individuals [39, 40]. Empowering employees can encourage risk-taking, innovation and initiative [41]. High levels of empowerment are also more likely to promote individual team members’ motivational states even when there are minor relationship conflicts within the team [42]. Such knowledge can be used to educate pharmacy students, pharmacists and pharmacy owners to empower their teams during the implementation of innovations such as professional services.

It is also worthwhile noting that the strategy category with the lowest PRP was ‘to communicate the change to stakeholders’. This is an interesting finding that conflicts with much of the literature around the importance of communication in a team environment [43]. When looking at the examples within this strategy category (Table 2), however, all strategies pertain to informing stakeholders of the changes that are happening, for example, inform the entire group of the change and objectives verbally, inform individuals of the change and objectives verbally, inform using a visual display such as poster and inform using a written document (email, letter, etc.). If one looks at the difference between this category and the categories with the highest PRP’s, these included ‘engaging stakeholders’, ‘equipping stakeholders’ and ‘empowering stakeholders’, rather than simply ‘informing stakeholders’. Challenges with providing information to others via methods such as email have been highlighted in previous research, such as ‘the absence of interpersonal clues’ [44]. In addition, ‘informing stakeholders’ indicates one-way communication and therefore may not adequately accommodate for deeper two-way discussions, active engagement or the ‘opportunity to interact and develop a shared understanding about the process they need to undertake to achieve their shared goals’ [45]. Pharmacy researchers have indicated the need for ‘improved engagement strategies to increase awareness and acceptance of innovations, promoting whole-team involvement within pharmacies to overcome time constraints’ [46].

When reporting on strategies used by change facilitators, it is important to recognise that change facilitators used a combination of strategies and that, even though some strategies were more effective than others, they were still used in combination with others. For example, to overcome ‘the inability to plan for change’, the most effective strategy predicted to resolve the barrier was to ‘engage stakeholders by creating ownership of the change’ which had a PRP of 84%; this, however, was closely followed with the strategy ‘equipping with training’ which had a PRP of 83%, and closely after that was ‘adapt area of focus to meet change needs’ which had a PRP of 81%. Change facilitators used all of these strategies in combination in order to successfully overcome the ‘inability to plan for change’. Change facilitators, therefore, must not isolate a change strategy and expect it to work by itself, but are encouraged to use a combination of strategies to tackle implementation barriers.

### Future application of this research

The challenge of evaluating facilitation strategies has previously been highlighted [47], with evaluation predominantly focusing on implementation or patient outcomes [20]. There is minimal focus on the granular strategies used by change facilitators during the implementation of innovation and the link between barriers and strategies [48]. In this study, change facilitators were given a tool in the form of an MS Excel Spreadsheet that enabled them to record implementation barriers discovered, facilitation strategies conducted and evaluation of the outcomes of their strategies. Such an approach can help change facilitators navigate implementation more systematically and collect effectiveness data that can be used during subsequent implementation studies to reduce implementation timeframes and increase adoption by stakeholders.

The data-driven, tailored facilitation approach used during this study can be applied to understanding common barriers to implementing innovation and the most effective change facilitation strategies to overcome these barriers in other industries outside of pharmacy.

Researchers in pharmacy practice need to further validate this tailored approach to ensure that implementation barriers uncovered during this study are consistent across community pharmacy and the effectiveness of the facilitation strategies is also consistent when implementing different innovations in community pharmacy.

Findings from this research can provide change facilitators, researchers and implementation teams with tailored strategies to overcome real-time barriers during the implementation of innovations in community pharmacy and other healthcare industries.

### Limitations

For increased predictive accuracy, data mining techniques require larger data points. The decision tree approach was determined as providing the best accuracy given the limited number of data points collected by the end of the 2-year programme. As only 19 pharmacies were involved in the change programme, the degree of implementation of services in the participating pharmacies is not necessarily a true representation of the pharmacy industry. One can argue that such teams showed a distinct level of innovation and early adoption that may not be a true reflection of the pharmacy industry. The use of a pre-determined and pre-defined list of implementation barriers aided in minimising coding inaccuracies. In addition, the collection of such information from 6 different change facilitators ensured that there were multiple independent coders of the implementation barriers. In terms of the facilitation strategies, however, limitations also apply to how the collected data was interpreted and coded by the research project manager, which is an inherent limitation to qualitative research. Limitations include research quality that is heavily dependent on the individual skills of the researcher and more easily influenced by the researcher’s (and change facilitators’) personal biases and idiosyncrasies [49].

## Conclusion

Results from the current study have provided a better understanding of implementation barriers in community pharmacy with the predominant barriers identified during this study, being an inability to plan for change, lack of internal supporters of the change and a lack of knowledge and experience regarding the change. The predicted effective strategies include those that aim to empower pharmacy teams to develop objectives and solve problems, engage teams by creating ownership, and equip teams with training, whilst the strategies with the lowest predictive resolution percentage are those relating to informing stakeholders of the changes. This insight not only shapes the way change facilitators can more effectively implement tailored facilitation strategies, it can also be used during future implementation projects for more efficient real-time implementation of innovation.

## Supplementary Information

**Additional file 1.** The 36 implementation barriers recorded by change facilitators, their frequency and definitions.

**Additional file 2.** The change facilitation strategies conducted by change facilitators, grouped into the 16 categories.

**Additional file 3.** The data analysis report by the Advanced Analytics Team at the University of Technology Sydney.

## Data Availability

The data that support the findings of this study are available from the corresponding author, but restrictions apply to the availability of these data, which were used under license for the current study, and so are not publicly available. Data are however available from the authors upon reasonable request and with permission of the Pharmaceutical Society of Australia.

## Abbreviations

PARiHS: Promoting Action on Research Implementation in Health Services HDP Health Destination Pharmacy
TDF: Theoretical Domains Framework
CFIR: Consolidated Framework of Implementation Research
TICD: The Integrated Checklist of Determinants of practice
PSA: Pharmaceutical Society of Australia
PRP: Predictive resolution percentage
RF: Random forest
