# Supplementary material for: Data-driven approach for tailoring facilitation strategies to overcome implementation barriers in community pharmacy
Source: Implement Sci. 2021 Jul 19;16:73. doi: 10.1186/s13012-021-01138-8 (PMC8290596; doi:10.1186/s13012-021-01138-8)
Supplement: Supplementary file 1 — Additional file 1. The 36 implementation barriers recorded by change facilitators, their frequency and definitions. [file 13012_2021_1138_MOESM1_ESM.docx]

**Additional file 1 | The description of the implementation factors uncovered by the Change Facilitators during the two-year study in pharmacy practice.**

| **IMPLEMENTATION FACTOR** | Frequency of appearance (n=1131) | **DESCRIPTION OF IMPLEMENTATION FACTOR** |
| --- | --- | --- |
| Ability to plan change [[1]](https://paperpile.com/c/gO60uw/rCpSt) | 184 | Planning associated with implementation of the change program*. |
| Internal supporters and opponents of the change [[1]](https://paperpile.com/c/gO60uw/rCpSt) | 128 | Support provided by the pharmacy staff members for the implementation of the program.  *(Ex.: help from peers or co-workers, time needed to provide the service, etc.)* |
| Knowledge/ experience [[2]](https://paperpile.com/c/gO60uw/o9LIt) | 84 | The extent to which the targeted individuals have skills, knowledge and experience that they need to adhere. |
| Monitoring and feedback [[1]](https://paperpile.com/c/gO60uw/rCpSt) | 61 | The extent to which monitoring and feedback are needed at organisational level and available to sustain necessary changes. |
| Individual alignment with the change (compatibility) [[3]](https://paperpile.com/c/gO60uw/emgs) | 49 | The degree of tangible fit between meaning and values attached to the intervention by involved individuals, how those align with individuals’ own norms, values and perceived risks and needs. |
| Objectives and feedback [[1]](https://paperpile.com/c/gO60uw/rCpSt) | 46 | The degree to which implementation objectives have been defined, communicated and achieved by the members of the pharmacy. (*E.g.: Objectives for the provision of the program, objectives set to solve detected barriers, target number of patients).* |
| Time^^^ | 43 | Amount of time devoted to implementation of the change. |
| Team communication^^^ | 42 | Type, quantity, communication flow between the pharmacy’s staff around the program. |
| Priority perception[[1,3]](https://paperpile.com/c/gO60uw/emgs+rCpSt) | 41 | Perception shared by the pharmacy’s workers about the importance of the implementation of the program in their own pharmacy. |
| Awareness of benefits of the change [[1]](https://paperpile.com/c/gO60uw/rCpSt) | 39 | Level up to which the benefits of providing the program are seen by individuals  *(E.g.: Improvement in results regarding health, higher satisfaction, increase in patient fidelity, improving relationships with physicians, “pull factor”, etc.*) |
| Teamwork^^^ | 38 | Abilities of the pharmacy’s staff to work together as a group. |
| Workflow (Team processes) [[1]](https://paperpile.com/c/gO60uw/rCpSt) | 33 | Way in which the pharmacy’s activities are divided and coordinated amongst its staff, including how pharmacy tasks are structured, how they are performed, in what order, how they are synchronised and how this affects the provision of the program. |
| Perceived complexity of the change [[3]](https://paperpile.com/c/gO60uw/emgs) | 29 | Difficulty perceived for the implementation of the program in the pharmacy, described by the duration, objectives and strategies required within the program. |
| Awareness of the change [[1]](https://paperpile.com/c/gO60uw/rCpSt) | 29 | The extent to which the participants are aware of and familiar with the recommendations of the program. |
| Emotions towards the change [[1]](https://paperpile.com/c/gO60uw/rCpSt) | 28 | The extent to which emotions affect adherence e.g. enthusiasm, frustration, cognitive overload, tiredness, regret. |
| Customer needs [[1]](https://paperpile.com/c/gO60uw/rCpSt) | 27 | Real or perceived needs and demands of the patients and whether they are met by the change being implemented. |
| Resources availability [[1]](https://paperpile.com/c/gO60uw/rCpSt) | 26 | Availability, quality & quantity of resources at the pharmacy to cater for customer needs. |
| Knowledge of own practice [[1]](https://paperpile.com/c/gO60uw/rCpSt) | 24 | The extent to which the targeted healthcare professionals are aware of their own practice in relationship to recommended practice. |
| Structural characteristics [[3]](https://paperpile.com/c/gO60uw/emgs) | 22 | Pharmacy design, age, size and maturity in relation to the provision of the program. |
| Leadership engagement [[3]](https://paperpile.com/c/gO60uw/emgs) | 22 | Commitment, involvement, capability and responsibility of the head of the pharmacy towards implementing the program. |
| Financial incentives (service profitability [[1]](https://paperpile.com/c/gO60uw/rCpSt) | 19 | The extent to which individuals have financial incentives or disincentives to adhere (e.g. ability to earn a profit from the program) |
| Self- efficacy [[1,3]](https://paperpile.com/c/gO60uw/emgs+rCpSt) | 19 | Provider’s self-beliefs to achieve the objectives established to provide and implement the program. |
| Culture [[3]](https://paperpile.com/c/gO60uw/emgs) | 17 | Expectations and shared values of all the pharmacy’s members. |
| Physicians’ knowledge and beliefs^^^ | 17 | Perception and knowledge of physicians on the necessity of providing the program through pharmacists. |
| Individual characteristics [[3]](https://paperpile.com/c/gO60uw/emgs) | 13 | Qualities, features or personalities of the participants that will act as enablers or become barriers when implementing the program. |
| External support [[1,3]](https://paperpile.com/c/gO60uw/emgs+rCpSt) | 11 | Measure to which a pharmacy receives the external support required for practice change. *(E.g.: Facilitator, clinical sessions organised by PSA.)* |
| Communication with patients^^^ | 10 | Participantⁿ skills when communicating with patients during the program. |
| Individual identification [[3]](https://paperpile.com/c/gO60uw/emgs) | 9 | How individuals perceive the organisation and their relationship, job satisfaction and degree of commitment with that organisation. |
| Resource use by staff^^^ | 6 | Level of use of the adequate bibliographical / technological resources to implement the program. |
| Individual stage of change [[3]](https://paperpile.com/c/gO60uw/emgs) | 3 | Stage at which each participant sits in relation to the evolution and progress of the program over time *(Precontemplation🡪 Contemplation 🡪 Preparation 🡪 Integration 🡪 Maintenance).* |
| Readiness indicators [[3]](https://paperpile.com/c/gO60uw/emgs) | 3 | Indicators inside of the pharmacy that show its commitment to the implementation of the program. |
| Patient awareness and perceptions^^^ | 3 | Patient awareness of the change and availability of marketing material to capture the patients’ attention. |
| Relationship with surrounding physicians [[3]](https://paperpile.com/c/gO60uw/emgs) | 2 | Working relationships established between the pharmacy and its pharmacists and physicians within its surroundings. |
| General business planning ^ | 2 | The extent to which leadership/team alter the strategic direction of the business. |
| Recruitment ability^^^ | 1 | Participants’ capability to enrol patients onto the various professional services. |
| Competitor pressure^ | 1 | The extent to which competition is affecting the pharmacy. |

^ Implementation factors relating to the program or suggested by the Change Facilitators.

ⁿ Participants refers to those pharmacy teams who participated in the program, this includes pharmacists, pharmacy technicians, pharmacy assistants and other team members.

* The program refers to the two-year ‘Health Destination Pharmacy’ program.

**References**

1. [Flottorp SA, Oxman AD, Krause J, Musila NR, Wensing M, Godycki-Cwirko M, et al. A checklist for identifying determinants of practice: A systematic review and synthesis of frameworks and taxonomies of factors that prevent or enable improvements in healthcare professional practice [Internet]. Vol. 8, Implementation Science. 2013. Available from:](http://paperpile.com/b/gO60uw/rCpSt) <http://dx.doi.org/10.1186/1748-5908-8-35>

2. [Michie S, Johnston M, Abraham C, Lawton R, Parker D, Walker A, et al. Making psychological theory useful for implementing evidence based practice: a consensus approach. Qual Saf Health Care. 2005 Feb;14(1):26–33.](http://paperpile.com/b/gO60uw/o9LIt)

3. [Damschroder LJ, Hagedorn HJ. A guiding framework and approach for implementation research in substance use disorders treatment. Psychol Addict Behav. 2011 Jun;25(2):194–205.](http://paperpile.com/b/gO60uw/emgs)
