# Supplementary material for: Data-driven approach for tailoring facilitation strategies to overcome implementation barriers in community pharmacy
Source: Implement Sci. 2021 Jul 19;16:73. doi: 10.1186/s13012-021-01138-8 (PMC8290596; doi:10.1186/s13012-021-01138-8)
Supplement: Supplementary file 3 — Additional file 3. The data analysis report by the Advanced Analytics Team at the University of Technology Sydney. [file 13012_2021_1138_MOESM3_ESM.docx]

**Additional File 3**

Paper: **Data-driven approach to tailoring change facilitation strategies to overcome implementation barriers | A study in community pharmacy**

Supplementary file authors:

Katarzyna Musial,

Simon Kocbek

Any questions regarding this supplementary file, please contact Professor Katarzyna Musial via email on [katarzyna.musial-gabrys@uts.edu.au](mailto:katarzyna.musial-gabrys@uts.edu.au)

1. **Context Understanding and Problem Statement**

This project analyses a real-world dataset from the pharmacy industry. Pharmacy practice is a significant component of the healthcare system in Australia and is constantly changing.

During the implementation of innovation in pharmacy practice, many barriers may arise. Hence, how to overcome these barriers becomes quite important.

The dataset’s provider requires a recommendation system of strategies for barriers that arise. The practical data has many limitations which causes difficulties for the analysis. It is common that datasets collected from real businesses have problems. In our case, the dataset is insufficient and unbalanced. We reviewed many related work to find out the best way to overcome problems arised from analyzing this kind of dataset.

The report focuses on a certain case, so the method may not be suitable in other cases. While the data type may not be the same as the data type in other cases, so the method tools (in this case Random Forest) may not be suitable in other cases. There are other method tools that can help improve the performance in further analysis.

The project aims to analyse the data to find the relationship between barriers and strategies. If there is a barrier, what strategies can resolve it. The project also aims to highlight the strategies with high effectiveness. Finally, the project tries to use classification methods to find which may be the best strategy corresponding to a certain barrier.

1. **Data Understanding and Data Preparation**
   1. **Data description**

The dataset consists of 1131 data points collected from six change facilitators across Australia during a 2-year pharmacy change program. The data included different types of barriers that prevent the change facilitators from implementing professional pharmacy services in the pharmacy setting.

In the given dataset, strategies have been separated into three categories from brief to detailed. The primary level of categorisation of the strategies has four phases: (i) Awareness of the need to change, (ii) Preparing participants for change, (iii) Planning for managing change, and (iv) Monitoring of change. The secondary level of categorisation of the strategies has 16 facilitation categories, and the tertiary level of categorisation of the strategies has 111 different types of strategies (additional table 2). These have been used by facilitators to deal with appropriate barriers. The dataset also provides the result of whether the issues have been resolved. Table 1 provides a summary of the data set that was provided to us for analysis.

**Table 1. The given data set was provided**

| **Attribute** | **Description** |
| --- | --- |
| Pharm no. | Integers from 1-19. Each different number represents a different pharmacy facility that provides the data. |
| Barrier code | Possible barriers to why the barriers are appearing. |
| Strategy (P.S.T) code | Each strategy code represents a strategy that is applied to solve a given barrier. Strategies are sorted by three categories, Primary strategy category (P), Secondary strategy category (S) and Tertiary strategy category (T). |
| Primary strategy category (P) | The first category of strategies. |
| Secondary strategy category (S) | The second category of strategies. |
| Tertiary strategy category (T) | The third category of strategies. |
| Result achieved | Whether the strategy resolved the barrier or not. |
| Visit No. | The visit in which this strategy was conducted. |

- 1. **Data understanding**

To get a preliminary understanding of the given dataset, we chose to analyze the dataset from a statistical perspective. Statistics is a mathematical description of a data collection, which could potentially present hidden patterns of attributes. In our case, statistical analysis could help us to know the distribution of the data set.

Due to the classified structure, there are three categories of the strategies. In the first category, there are only 4 different values, which have few logical relationships for analysis. At the same time, in the data description, the amount of data in the tertiary category of strategies is too large, with 111 different strategies, some of which only appear once.

The secondary category of strategies appears dozens of times, and seems to have relationships with the barriers column. This why the secondary category was chosen for analysis.

**SMALL DATA SET**

The data contains 1131 data points, the amount of the tertiary level of categorisation of the strategies is 111. Strategies within the tertiary categories vary in numbers from 1-10 records only.

**UNBALANCED DATA**

The value in the target attributes shows unbalanced distribution. The ‘Unresolved’ value only occupies 159 records of the whole, about 14%, while the rest are all ‘Resolved’ occupying about 86% of the whole data. The unbalanced distribution in the target column leads to low efficient prediction. In the software, the model will simply predict all the results as ‘Resolve’, the major value of the attribute, and get a high accuracy rate as well.

1. **Modelling and Evaluation**
   1. **Statistical analysis**

As it was shown in the data understanding section the best level to analyse the strategies is the secondary category of strategies. The main purpose of analyzing the secondary category is to find which is the best strategy corresponding to each barrier. For each barrier, there are many strategies used, while the Resolution Percentage in the result column may not be the same. The best strategy should have the highest Resolution Percentage, and also needs to be used in most cases. The first step is to filter and concentrate on one barrier. Then, the second step is to have a look at the Resolution Percentage of each strategy. In the end, the report collects and generates a table of those strategies which have the highest Resolution Percentage and are used in most cases.

The table generated seems good for business. If there is a barrier happening in a new case, the Change Facilitator can use the table to find what is the strategy suggested to deal with it. The further work can make the result of the table into the system of users, once the Change Facilitator chooses or imports the barrier or barriers faced, the system can output the recommended strategy to them.

While in the process of selecting and comparing the secondary category of strategies, the analysis encounters some problems, which are very common in real world business cases. In the generated table, it can be seen that many results do not meet the expectation.

One problem is that there are many strategies that have similar Resolution Percentages in many cases. For example, for barrier ‘7’, it has 65 records, while many strategies all have 100% Resolution Percentage like ‘2.1’, ‘2.4’, ‘2.5’, ‘3.2’, ‘3.5’, ‘3.6’. In this situation, the analysis can not point out which of them is the best for the recommendation, so the table keeps them all. In the further work, one option is to choose ‘3.5’ which has the most records, 23 times of the whole 65 records. Another option is to use a method to find which of them is better than others.

Another problem is that the chosen strategies only occupied a small percentage of the whole records. For example, for barrier ’24’, the strategy ‘2.4’ has the highest Resolution Percentage, while others are all unsatisfied. The strategy ‘2.4’ only has 6 records of the whole 17 records, about ⅓, which may not be representative. In this situation, the analysis can hardly promote the result.

In some cases, strategies with a low Resolution Percentage occupy a high percentage of the whole record, while each of the other strategies with high Resolution Percentage only occupy a low percentage. For example, in barrier ‘3’, it only has one record, strategy ‘3.4’. But the strategy has only one Unresolve result. In this situation, the analysis lacks recommendation, but only supports the strategy ‘3.4’ and its result for reference. The same problem occurs many times in the dataset. Some strategies have a low Resolution Percentage, but occupy the majority rate of the records.

While providing recommendations based on statistical analysis, there is a controversial way of choosing strategies in some cases.

**Table 2. Example of challenges associated with statistical analysis**

| **Barrier Code** | **Secondary Strategy Category** | **Sample amount/ solved amount** |
| --- | --- | --- |
| 15.0 | 3.5 | 56/59 |
|  | 2.1 | 10/10 |
|  | Others | 14/15 |
|  | Total | 80/84 |

As shown in table 2, 84 cases of dealing with the barrier of barrier code 15 are recorded. While strategy 3.5 appears 59 times with 56 times solved, strategy 2.1 appears 10 times with 10 times solved. So the Resolution Percentage is 94.9% vs 100%. Although strategy 2.1 seems to have a better Resolution Percentage, its sample amount is far less than strategy 3.5.

Therefore, it’s hard to say which strategy is better. We believe that there could be a function curve that describes the pattern of how people in the pharmacy industry choose regarding the number of occurrences and the Resolution Percentage.

An assumption is that we can gather the preference information from people in the pharmacy industry and generate a function curve based on the preference information. In this case, we can give the best recommendation based on the sample amount’s scale and the Resolution Percentage ratio.


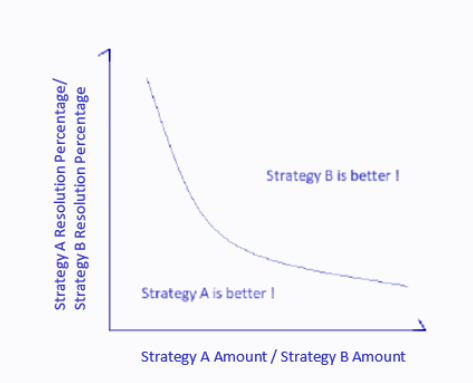


*Figure 2 Resolution Percentage vs. Strategy amount*

As shown in Figure 2, the X-axis is the ratio value between the amount of strategy A and B, while the Y-axis is the ratio value between the Resolution Percentage of strategy A and B. The curve is behalf of the case that strategy A is equal to strategy B. If the point is below the curve, strategy A is better than strategy B. If the point is above the curve, strategy B is better.

**Conclusion for statistical analysis**

The generated table from statistical analysis basically meets the requirement. The pharmacy managers can use the table to deal with new barriers of events. While there are some drawbacks in the table mentioned above, these issues may not barrier problems but may make users confused about the way to deal with new barriers. The next step of the analysis focuses on the problem of how to select the best strategy if many strategies have the same Resolution Percentage.

- 1. **Random forest**

As the problems mentioned in the statistical analysis, the report tries to find the best strategy for many barriers. The research chooses several methods to achieve the goal. One way is to predict the result of each strategy and barrier to find which has a better result, so when in the next time, in a new barrier, if change facilitators use this strategy, they can have a high rate to overcome the barrier. The best way is to use a classification analysis method to make a prediction.

In business, companies often use the Random Forest to make predictions. Random forest is one of the best classification methods, and it is much better than the Decision Tree method. Random forest combines great numbers of analysis trees trained randomly and equally from the dataset. The theory of building a random forest contains training and testing steps. In the training step, the method uses the majority of the dataset to build the algorithms, which have a high relationship to target result. In this step, it builds large numbers of trees and then evaluates separately and averages them to compute the estimate. After that, the method uses the rest of the data to test the workflow, and find the accuracy of the prediction. The best advantage of using random forest is that it is stable, which has less overfitting to other classification methods.

In a previous paper, the authors [(Khalilia et al. 2011)](https://paperpile.com/c/8S3KOW/9tIY) used the Random Forest to predict disease risk of individuals by analyzing their medical diagnosis. They also compared different methods and faced imbalanced data. The result met their expectation, and they predicted 8 disease categories. While, in another paper, Random Forest was used to predict business performance from small and Swiss companies[(Muller et al. 2017)](https://paperpile.com/c/8S3KOW/TPeb). The sample is more related to how to choose strategies. The content and background of these papers are similar to this case. It identifies the validity and reliability of using the Random forest in this analysis.

**WORKFLOW**


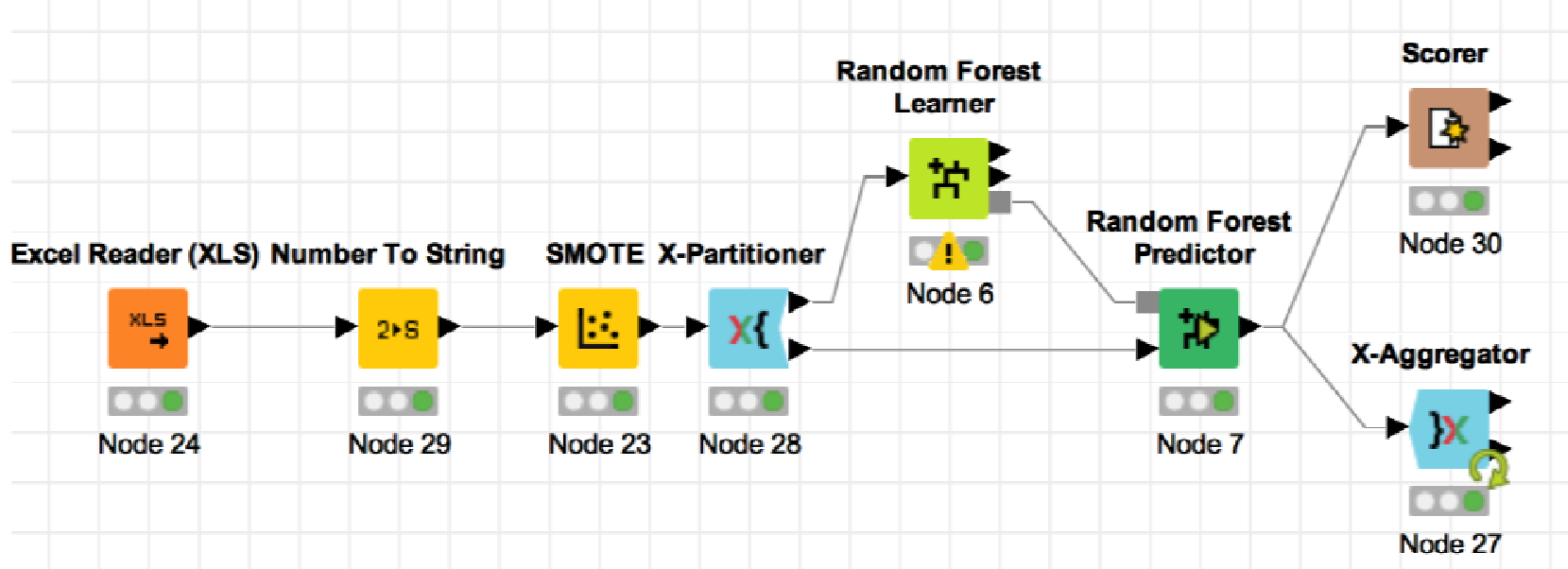


*Figure 3 Workflow diagram 1 for Random Forest analysis*

**BALANCING THAT DATA**

As it was shown in the data understanding section, the classes are very unbalanced.

The first reason for the problem is that the Unresolved results only occupy a small part of the whole results. Bebarrier in the ‘result achieved’ column of original data, the value ‘Resolve’ occupied about 86%, 7 times Unresolved. The Random Forest method may recognize the prediction calculated as the majority of the original data set and ignore the importance of the minority part of the value in the target column.

This is a typical problem that happens in business, which is called unbalanced data or imbalanced data. The problem is prevalent in machine learning. Most machine learning classification algorithms are sensitive to unbalance [(Witten et al. 2016)](https://paperpile.com/c/8S3KOW/hc1w). An unbalanced dataset will bias the prediction model towards the more common class [(Glander 2017)](https://paperpile.com/c/8S3KOW/c76t). When a classification algorithm trained on such data is applied to a test data set that is also unbalanced, the classifier will produce a very optimistic accuracy estimate. Many articles show the problem rises attention of researchers. Cieslak et al. point out that it is necessary to generate new method based on existing algorithms to lower the effect of unbalanced data in their article, while other articles all apply new tools to fix the bias [(Cieslak and Chawla 2008)](https://paperpile.com/c/8S3KOW/v2tT). Ye and Rick discussed and supported several methods to fix the problems including resampling the training set, using K-fold Cross-Validation in the right way, ensembling different resampled datasets, resampling with different ratios and so on (Ye and Rick, 2015). In the analysis of Khoshgoftaar et al. , the authors tried to compare ensemble and data sampling which is better to fix the unbalanced data (Khoshgoftaar et al., 2015). The findings show that both Select-Bagging with Naive Bayes and Random Forest with 100 trees are recommended for imbalanced datasets.

**FINAL RUN**

The workflow for the final prediction is shown in Figure 4. All nodes about Cross-Validation has been removed. This is bebarrier the use of it is to make sure that while dividing the dataset into training part and test part, just in case of extreme cases, like a great part of training dataset is ‘Solved’. Since we are making real predictions on all combinations of barriers and strategies, we no longer need to cut the original dataset. Instead, all original dataset is imported as training dataset from the above Excel Reader (XLS) node. What we put into the below Excel Reader node is a new table we generated, with all possible combination of strategies and barriers listed. The final output from the Random Forest Predictor should tell us the detailed probabilities of each combination.


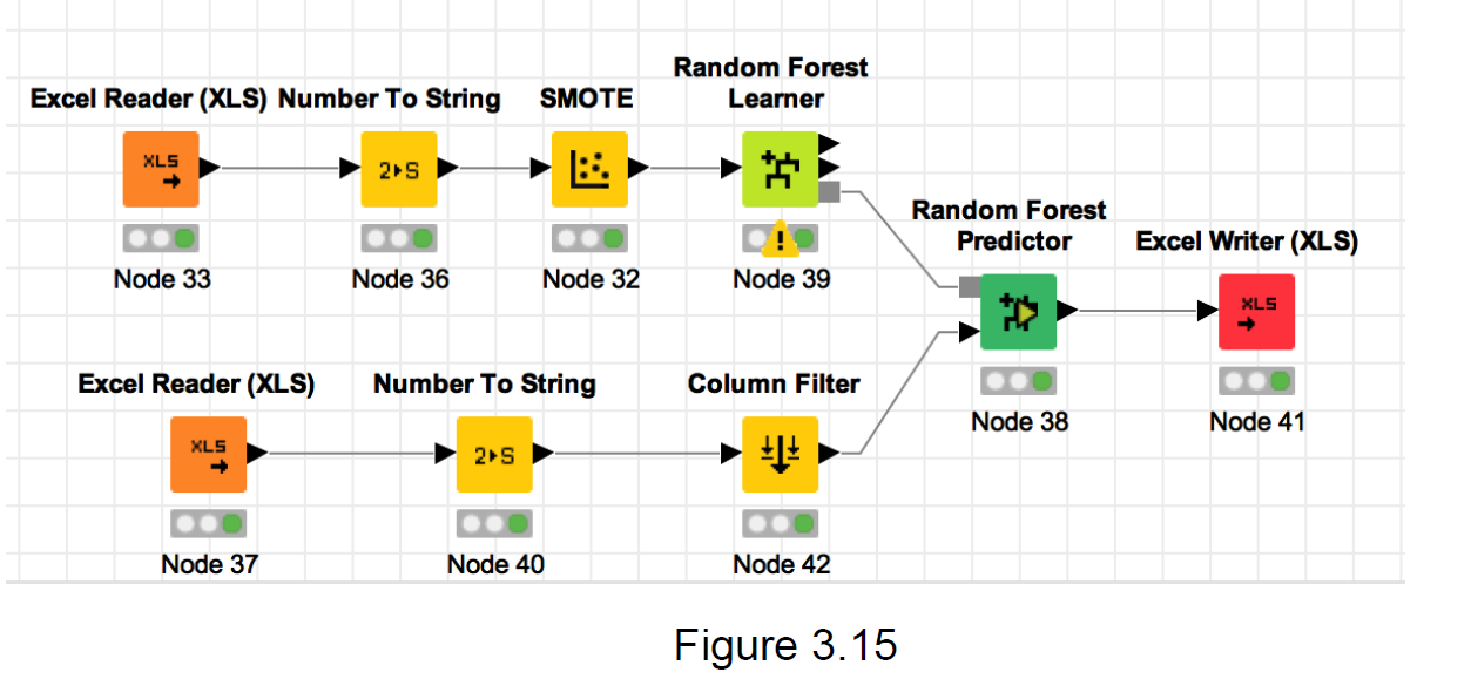


*Figure 4 Workflow diagram 2 for Random Forest analysis*

After using the model, the test uses a ‘’Excel writer’ node to output a result which contains all the results predicted and their possibilities.

In this case, their possibilities are between 0 to 1. There are both possibility rates of Resolved and Unresolved. If the possibility rate of Resolve is higher, so the strategy is better and suggested to the barrier. In the previous statistical table generated, there are many coordinated strategies. Though the result in the original data set is Resolve, the possible rate of the Resolve may not be 100%. The output can support a better explanation of each result in this way.

For example, in barrier A, strategy X and strategy Y have the same Resolution Percentage in the statistical table, 100%. The output from the model can show the possible rate of each record. The average of the possibility rate of Resolution can be calculated. If the Predictive Resolution Percentage of strategy A is 99% , higher than strategy B, so the output points out strategy A is better.

1. **Discussion and Recommendations**

Since the provided dataset is quite insufficient, we decided to keep the initial recommendations while providing recommendations based on the modelling as well. Therefore, we provided two kinds of recommendations.

**RECOMMENDATION BASED ON STATISTICAL ANALYSIS**

The recommendation by statistical analysis provides the best-recommended strategy based on the given dataset. The dataset is rather sufficient, although it can provide recommendations, it doesn’t provide a detailed recommendation (Table 2). It only presents which strategies appear more in the dataset with their Resolution Percentage. In this case, the recommendation is not clear.

**RECOMMENDATION BASED ON RANDOM FOREST ALGORITHM**

The recommendation based on Random Forest Algorithm provides a better perspective to analyze the dataset. In recommendation based on statistical analysis, different strategies recommended for one barrier may have the same Resolution Percentage- which leads to a dilemma, which one is the actual better one. The application of Random Forest Algorithm utilises hidden relations between ‘Resolved achieved’ and other and contributes to making ‘prediction’ of the ‘Resolved achieved’ attribute. In this case, the values of other attributes are fully utilized. Differences between those strategies with the same Predictive Resolution Percentages appear. e.g. In the first recommendation, barrier code 11 has two recommended strategies (Table 3). Strategy 2.5 has more sample amounts with a slightly low Resolution Percentage. In recommendation based on Random Forest Algorithm, it shows that with the contribution of other attributes, strategy 2.5 actually has a higher Predictive Resolution Percentage (Table 3), meaning strategy 2.5 is the better choice.

**Table 3. Results using statistical analysis versus results using Random Forest**

| **Barrier Code** | **Secondary Strategy Category** | **Sample amount/ solved amount** | **Resolution Percentage**  **(using statistical analysis)** | **Predictive Resolution Percentage (using Random Forest)** |
| --- | --- | --- | --- | --- |
| 11.0 | 2.5 | 16/18 | 88.89% | 87.72% |
|  | 2.1 | 9/10 | 90.00% | 85.65% |

In this project, we provided two kinds of recommended strategies using two ways of analysis. The recommendation from statistical analysis cannot provide a precise recommendation in some cases, a possible solution is to conduct a satisfaction curve based on user research, so that the recommendations could be narrowed down to one. Since we don’t have the data for this, this is a suggestion for future research.

Another solution, which is our second recommendation, is using a Random Forest Algorithm to generate a more precise Predictive Resolution Percentage for each strategy according to each barrier. This might be the potentially best way to solve this problem. However, due to the insufficient data volume, there are some cases that we still cannot provide precise recommendations, but the result is better than just applying statistical analysis.

**Reference**

Shearer, C., 2000. The CRISP-DM model: the new blueprint for data mining. Journal of data warehousing, 5(4), pp.13-22.

Justin Stoltzfus, How might companies use random forest models for predictions?, viewed on 10 October 2018, <https://www.techopedia.com/how-might-companies-use-random-forest-models-for-predictions/7/32995>.

Müller, D., Te, Y.-F. & Jain, P. 2017, 'Predicting business performance through patent applications', Big Data (Big Data), 2017 IEEE International Conference on, IEEE, pp. 4159-64.

Khalilia, M., Chakraborty, S. & Popescu, M. 2011, 'Predicting disease risks from highly imbalanced data using random forest', BMC medical informatics and decision making, vol. 11, no. 1, p. 51.

Statistics, viewed on 10 Oct 2018, <https://en.wikipedia.org/wiki/Statistics>.

Witten, I.H., Frank, E., Hall, M.A. & Pal, C.J. 2016, Data Mining: Practical machine learning tools and techniques, Morgan Kaufmann.

Shirin Glander, 2017, Dealing with unbalanced data in machine learning, Github, viewed on 16 Oct 2018, <https://shiring.github.io/machine_learning/2017/04/02/unbalanced>.

Cieslak, D.A. & Chawla, N.V. 2008, 'Learning decision trees for unbalanced data', Joint European Conference on Machine Learning and Knowledge Discovery in Databases, Springer, pp. 241-56.

Ye Wu & Rick Radewagen, 2017, 7 Techniques to Handle Imbalanced Data, IE Business School.

Khoshgoftaar, T.M., Fazelpour, A., Dittman, D.J. & Napolitano, A. 2015, 'Ensemble vs. data sampling: Which option is best suited to improve classification performance of imbalanced bioinformatics data?', Tools with Artificial Intelligence (ICTAI), 2015 IEEE 27th International Conference on, IEEE, pp. 705-12.
